# Advances towards circular economy policies in the EU: The new Ecodesign regulation of enterprise servers

**DOI:** 10.1016/j.resconrec.2019.104426

**Published:** 2020-03

**Authors:** Laura Talens Peiró, Davide Polverini, Fulvio Ardente, Fabrice Mathieux

**Affiliations:** a Beatriu de Pinós at Sostenipra, Institut de Ciencia i Tecnologia Ambientals (ICTA-UAB), Universitat Autònoma de Barcelona, 08193 Cerdanyola del Vallès, Barcelona, Spain; b European Commission, DG Internal Market, Industry, Entrepreneurship and SMEs, Brussels, Belgium; c European Commission, Joint Research Centre (JRC), Directorate Sustainable Resources, Ispra, Italy

**Keywords:** Circular Economy (CE), Ecodesign, Enterprise servers, Policymaking, Critical raw materials (CRMs), Material efficiency

## Abstract

The concept of a circular economy has been widely accepted by governments and industries. In Europe, the European Commission adopted the Circular Economy package in 2015. The Ecodesign Directive has been identified as one of the most suitable legislative tools for achieving some of the objectives in the package because it has the potential to translate the circular economy principles into specific product material efficiency requirements. This paper applies the Ecodesign policy process to “enterprise servers” to illustrate how circular economy strategies can be implemented by European product policies. Indeed, the paper introduces a potential novel approach to “operationalize” circular economy principles in product policies. The evolution of the material efficiency requirements for a more circular economy is described up to their final formulation, which is the one in the published Ecodesign regulation. This legal act includes requirements on design for disassembly, firmware availability, data deletion, and presence of critical raw materials. The process for enterprise servers has been successful as the early discussions between stakeholders, policymakers and experts, supported by appropriate metrics along an iterative debate, comes to the publications of material efficiency requirements in a regulation. This study represents a 'first-of-a-kind' experience, and sets precedents for the development of similar requirements for other product groups.

## Highlights

• An approach to developing material efficiency requirements is illustrated.
• It uses product data and quantitative material efficiency indicators.
• It was “operationalized” on the basis of lessons-learnt during the policy processes.
• Circular economy aspects are identified, analysed, and discussed thoroughly.
• The timely involvement of key stakeholders and experts was crucial.

## 1 Introduction

Discussions on the Circular Economy (CE) concept itself date back to 1970s and 1980s so they are not new. Stahel and Reday-Mulvey, 1976 described a vision of an economy in terms of loops and its impact on job creation and competitiveness (Stahel and Reday-Mulvey, 1981). In the late 1980s, other authors initiated a discussion on the importance of closing material loops in industrial processes (Ayres, 1989; Frosch and Gallopoulos, 1989). Indeed, the Ellen MacArthur Foundation, one of the organisations that contributed the most to spreading the concept of CE, considers that CE is deep-rooted in several so-called schools of thought: cradle to cradle, the performance economy, biomimicry, industrial ecology, natural capitalism, the blue economy, and regenerative design (The Ellen MacArthur Foundation, 2019). Several definitions of CE have been suggested by a great many organisations in recent years (Kirchherr et al., 2017). CE is most frequently depicted as a combination of reduction/reuse/recycling activities, potentially linked to the sustainable development concepts but expressed with a large variety of definitions that reveals heterogeneity in the understanding of it (Kirchherr et al., 2017).

At EU level, a broad consensus on energy efficiency policies in the last decade has helped the attainment of ambitious energy efficiency targets in Europe (European Commission, 2010a). Now, especially after the publication of the EU Circular Economy Action Plan (EU CEAP) adopted by the European Commission (EC) in December 2015, the efficient management of materials is becoming an area of key focus (European Commission, 2015a). The EU CEAP consists of legislative proposals on waste and an action plan covering the whole life cycle of products and materials. The focus of the EU CEAP is broader than simple waste management as it aims to increase material efficiency and close loops by improving reuse and recycling. The use of product life cycle strategies in product policies is also gaining momentum outside the EU (Ometto and Filho, 2006).

Although aspects of CE (such as reparability, durability, upgradability, recyclability, or the content of certain materials or substances, including reuse and recycled content) have been discussed in the literature, their implementation in mandatory regulations has been very limited (Dalhammar et al., 2014; Bundgaard et al., 2017). Indeed, the publication of the EU CEAP represents an initial commitment to systematically examining CE aspects in future mandatory legislation, especially as part of the EU Ecodesign Directive (European Parliament and the Council of the European Union, 2009). Action 1 of the EU CEAP states that the EC will “emphasise CE aspects in future product requirements under the Ecodesign directive” (European Commission, 2015a). Before that, only a few examples of material efficiency requirements were implemented in European Ecodesign Regulations, mainly concerning the communication of general information to end-of-life operators. Examples include the declaration of the content of mercury in televisions (Dalhammar et al., 2014; Bundgaard et al., 2017). Indeed, the publication of the EU CEAP represents an initial commitment to systematically examining CE aspects in future mandatory legislation, especially as part of the EU Ecodesign Directive (European Parliament and the Council of the European Union, 2009). Action 1 of the EU CEAP states that the EC will “emphasise CE aspects in future product requirements under the Ecodesign directive” (European Commission, 2015aa). Before that, only a few examples of material efficiency requirements were implemented in European Ecodesign Regulations, mainly concerning the communication of general information to end-of-life operators. Examples include the declaration of the content of mercury in televisions (European Commission, 2009b) and lamps, water consumption in washing machines (European Commission, 2010b), and the provision of generic information for disposal of electrical motors and circulators (European Commission, 2009c). Only a few of the most advanced Ecodesign regulations include material efficiency requirements. One of them is the durability requirements of some critical parts in vacuum cleaners, namely the hose and the motor (Commission, 2013). In lamps, durability aspects are measured by the lumen maintenance factor as well as a number of other factors such as the number of switching cycles before failure, premature failure rate, and for some lighting products, colour rendering (European Commission, 2009a; Maitre and Dalhammar, 2016). In both cases, the availability of test methods and technical standards to ensure the enforceability of the regulation has been crucial (European Committee for Standardization (CEN), 2013; Bobba et al., 2016).

Besides these regulations, following the recommendations of the “Roadmap to a Resource Efficient Europe” Communication (European Commission, 2011), the EC Joint Research Centre has published several studies discussing potential material efficiency measures for several example products that could be implemented in European product policies, including Ecodesign requirements for electronic displays (Ecodesign and EU Ecolabel) (Ardente and Mathieux, 2014a,b; Ardente et al., 2015; Bobba et al., 2016; Talens Peiró et al., 2017; Ardente et al., 2018).

Establishing compulsory minimum requirement on material efficiency has been a challenge for several reasons, including: the lack of discussion of material efficiency concepts during the policy debate, the absence of appropriate metrics (commonly accepted material efficiency assessment methods, e.g., in standards), and the related problems of verifying requirements, the variability between product groups, and diverging interests of stakeholders along the value chain. This paper illustrates a method that was developed to cope with these challenges during the formulation of material efficiency requirements for enterprise servers. First, the paper discusses these challenges in detail (Section 2). Then, it analyses the current European Ecodesign policy process and how it could adapted to ‘operationalize’ CE principles for the development of EU product policies (Section 3). Section 4 describes the practical case-study experience on building CE requirements for the ‘enterprise servers’ product group, including the analysis of problems encountered and solutions adopted. Then, following a bottom-up approach, the paper analyses the lessons learnt and how these could be generalized into a novel lesson strengthening the development of CE measures under the EU Ecodesign regulation (Section 5). Finally, Section 6 summarizes the main findings and identifies further research that could strengthen the EU CEAP.

## 2 Challenges in developing circular economy measures in product policies

Three high-level policy goals concerning CE measures for products have been identified which are: reduction of the environmental impact, extension of the lifetime, and reduction of waste (Tecchio et al., 2017). These goals are to be achieved by implementing material efficiency requirements together with other environmental requirements in policies (e.g. climate change mitigation). Synergies among environmental policies can trigger greater change in the production systems and represent a “tipping point for industries: soon it will be too expensive to continue with business as usual” (Levänen and Eloneva, 2017).

There are several articles and studies explaining how including material efficiency requirements in product policies improves recovery and recycling of materials contained in end of life products, and so contributes to a more Circular Economy (Dalhammar et al., 2014; Bundgaard et al., 2017). For example, Ardente et al. (2015) discussed how the enforcement of features such as ‘design for dismantling’ through mandatory product policies such as the Ecodesign Directive could facilitate the end-of-life treatment of commercial refrigeration appliances and hence ease compliance with the waste legislation (Ardente et al., 2015).

The challenges to enabling a transition to CE in policy development can either be technical and practical in nature. One of the greatest technical challenges is to include concepts as reparability, durability, upgradability and recyclability in the product policy debate. Although these are sometimes ‘familiar’ to the general public, how much stakeholders understand can differ. Although conceptually different, these concepts are generally very similar and are even used interchangeably, for example, ‘design for disassembly’ and ‘design for dismantling’ (Vanegas et al., 2018). While ‘design for disassembly’ refers to the application of design principles to allow a non-destructive separation of the parts in a product, the ‘design for dismantling’ implies the use of a broader set of strategies, including the destructive separation of parts in a product that disables their re-construction. ‘Design for dismantling’ strategies therefore include reversible and irreversible operations that separate certain valuable components for their recovery and recycling.

The second technical challenge is the development of assessment methods, using scientifically robust metrics, to measure the performance of the product. One or more indicators related to CE aspects such as durability (Stamminger et al., 2018) or disassembly (Vanegas et al., 2018) can be defined. Furthermore, these metrics are fundamental to stipulating the correct material efficiency requirements in the legislation. Finally, assessment methods and metrics need to be unambiguously verifiable by market surveillance authorities before the product is launched on the market. For instance, once a requirement for ‘design for disassembly’ has been formulated, it is necessary to define the procedures to prove that a certain product fulfils the disassembly requirement.

The development of accurate metrics to measure certain aspects (e.g. durability) can be very different product by product, requiring the development of a dedicated testing method and potential standards. For instance, the durability of lamps is measured by the lumen maintenance factor whereas the durability of vacuum cleaners is assessed according to the fatigue life testing of the motor and hose. Products can also differ greatly in their end-of-life treatment. For wasted lamps, particular attention needs to be paid to regulated hazardous substances such as mercury, which requires a dedicated treatment line. Vacuum cleaners do not generally contain mercury or other regulated substances and therefore, they can be treated in shredders together with other small household appliances. Detailed knowledge of the pre-treatment and recycling processes of each product is crucial in defining a benchmark of current practices and to propose measures and targets for material efficiency, as demonstrated for the electronic displays product group (Ardente and Mathieux, 2014a).

Bundgaard et al. (2017) highlighted the need to expand the standardization work to define test methods and verification procedures concerning material efficiency requirements for the products to enhance an increasingly Circular Economy (Bundgaard et al., 2017). Tecchio et al. (2017) provided a first example of a framework to identify key material efficiency considerations relevant to product policies in support of sustainable engineering, and map out the generic and product specific standardisation requirements (such as appropriate metrics, tests, calculation procedures, reference tables, and structured templates for results) (Tecchio et al., 2017). Regarding the development of CE standards for policies, the standardization work currently being undertaken by the European standardization bodies under mandate M/543 (European Commission, 2015b) has been a big step forward worldwide. First horizontal standards (i.e. non-sector-specific, non-product-specific) concerning the provision of information on material efficiency aspects and on the use of critical raw materials (CRMs) were recently delivered, whereas additional standards are expected in 2019 (CEN-CENELEC, 2019). These standards will lay down basic principles for consideration when addressing aspects such as: the extension of the lifetime of products; the ability to separate components for reuse or for recycling; and the use of re-used components and/or recycled materials in products. Moreover, these standards will provide a complete set of definitions of key terms related to different CE strategies, and contribute to overcoming some of the challenges highlighted above.

The challenges of the so-called practical nature mainly refer to the development of the policymaking process itself and to external factors influencing stakeholders, and more directly the representatives of EU member states who have the final say on approving or amending regulations. This paper explains the development of the EU Ecodesign policy process, and how the EC can guide the process to guarantee a fair and objective discussion of material efficiency aspects from the beginning of the process. Some authors have examined the integration of material efficiency into the Ecodesign Directive in detail by ex-post analysis of several Ecodesign processes (Bundgaard et al., 2017; Gabarrell Durany et al., 2017). The recommendations improving the focus on material efficiency requirements in the Ecodesign Directive are: 1) material efficiency aspects need to be analysed in the early phase (i.e. in the preparatory studies) so that they have a chance of being considered during the political discussion; 2) the analysis of Energy-related Products (ErP) should include a specific focus on material efficiency and its indicators; 3) the involvement of stakeholders in CE during the preparatory study needs to be strengthened. Hinchliffe and Akkerman (2017) also argued that more time and effort should be devoted to material efficiency aspects so that its importance can be weighed up against energy efficiency and other parameters. The shortest ‘inner cycles’ described in the CE, namely maintenance, repair, and remanufacture have the greatest potential to generate the highest environmental and economic benefits as they help the products keep their value longer. A greater involvement of the original equipment manufacturer (OEMs) is crucial in defining requirements aimed at improving these stages. When defining regulatory measures on these stages, it has also been observed that the interests of stakeholders in CE strategies for products are often not aligned and so they diverge in some cases (Dalhammar, 2016). For example, some manufacturers claim that they are exclusively charged with the costs and the burdens related to an improved ‘design for recycling’ of the products, whereas these strategies mainly benefit waste treatment and recycling companies. Similarly, reuse and repair operators have interest in extending the lifetime of products as much as possible while some OEMs may be more oriented towards maximize the volumes of sales of new products rather than making them more durable. Some authors have highlighted the importance of involving waste treatment and recycling companies among the stakeholders in the early stages of the Ecodesign policy process and the formal consultation process of the Ecodesign Directive (Ardente et al., 2015). Experience shows that even though these companies might not have a special focus on improving the ‘inner cycles’ strategies like repair and maintenance, they tend to be highly collaborative. This collaboration in many cases facilitates the development of trial tests on samples of the product group under study, e.g., for the purpose of performing analyses of ‘design for disassembly’ which can particularly benefit the CE ‘inner cycles’ as well as the ‘outer cycles’.

**Fig. 1:**
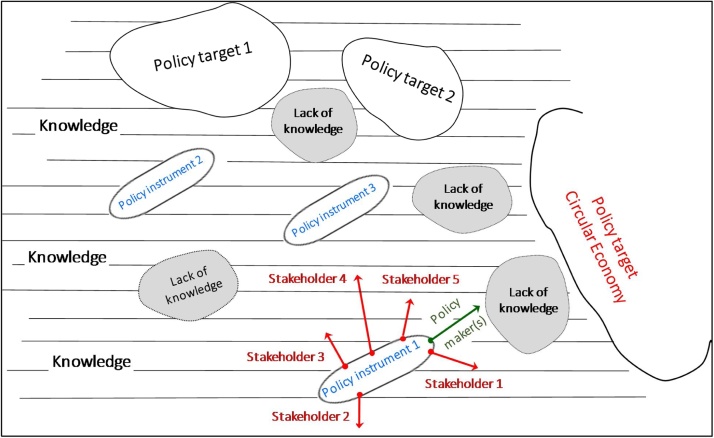
A conceptual representation of ‘policymaking’ using the ‘Travelling-by-ship’ metaphor.

Fig. 1 represents some of the practical challenges of the policymaking process via the metaphor of ‘travelling by ship’. Policymakers^1^ pursue various policy targets, one of them being current CE policies. To achieve the target, different policy instruments are available based on several inputs of information, like ships floating in a 'sea' of knowledge and moving towards the target islands. The effectiveness of the participatory modelling and stakeholder involvement has already been proved in various contexts; especially those related to resource management and to environmental impact assessment (Hedelin et al., 2017). In many cases, conflicting interests between diverse groups of stakeholders arise and, together with the areas of ‘lack of knowledge’, stand as barriers to the development of the policy and the achievements of the targets. In practical applications, different stakeholders contribute to policy instruments by having different interests (i.e. economics, business models, interest and power) as if they are 'rowers' pushing in different directions (towards different target signer) with different intensities. Generally, these barriers can be bypassed by additional scientific knowledge, evidences, data, and methods.

The policymakers are responsible for driving the policy instruments as if they are metaphorical 'steersmen' in this 'sea' of knowledge and interests, identifying the potential barriers to be overcome and conveying all of the various stakeholder ‘forces’ towards the target(s).

More in general, Dalhammar et al. (2014) found that the potential for setting rules for CE aspects in Ecodesign regulations is very dependent on the product group. Therefore, they argue that it is worth starting to work more coherently with material efficiency requirements under the Ecodesign Directive, and that it is wise to ‘advance slowly’ to avoid setbacks (Dalhammar et al., 2014). This paper follows the advice from Dalhammar et al. (2014) and analyses how CE strategies can be formulated as material efficiency requirements. The results of the case study on enterprise servers help understand the advances towards a more CE policy in EU product policies.

## 3 Methodology

The EU CEAP identified the European Ecodesign Directive as a key instrument in enhancing CE strategies in production and consumption systems in the EU (European Commission, 2015a). This Directive requires product manufacturers to improve the environmental performance of their products, typically by meeting minimum energy efficiency requirements as well as other environmental requirements such as water consumption or emission levels. It also aims to remove the worst-performing products from the EU market and help individuals and companies to reduce their utility bills (European Commission, 2016a). By acting at the level of the EU single market, it also avoids costs for business and consumers due to otherwise fragmented national environmental requirements. Finally, the implementation process of the Ecodesign Directive benefits from well-structured and broad support from stakeholders (Tanasescu, 2009), including industry, EU member states, consumer organisations, and non-governmental organizations (NGOs) because the transparent and regular consultation process in establishing implementing measures is much appreciated.

**Fig. 2:**
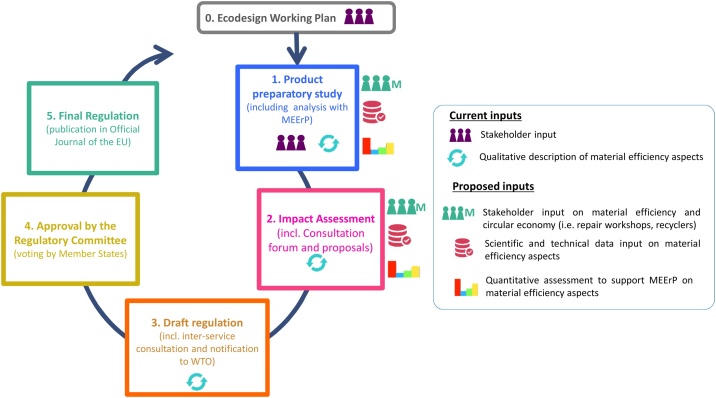
The proposed approach to 'operationalize' circular economy into product policy (Ecodesign) effectively.

In order to identify relevant Ecodesign measures for products, the EU developed the so-called ‘Methodology for Ecodesign of Energy-related Products’ (MEErP) (Kemna, 2011). This is a well-established method built on various technical and political steps (Fig. 2) that have been consolidated along its application for the assessment of many product groups (European Commission, 2018a). Please see Box 1 for a detailed description of the Ecodesign policy process.

Box 1 **The EU Ecodesign Directive policy process.**

The EU Ecodesign implementation process starts by analysing a number of product groups that are deemed relevant for the development of potential Ecodesign regulations, and the publication of a list of the most relevant products in the so-called ‘Ecodesign working plan’ (Step 0). In general, product groups are prioritized according to the potential environmental savings, to be attained if the Ecodesign regulations are enforced. The EU Ecodesign implementation process starts once a list of prioritized product groups is published.

The development of an EU Ecodesign regulation for a product group can be conceptualized into five steps (see coloured blocks in Fig. 2). The preparatory work (Step 1) prior to any Ecodesign policy measure is a complex exercise, which entails technical, procedural, and legal steps in accordance with a well-defined procedure. The analyses carried out in the preparatory studies are based on the application of the ‘Methodology for Ecodesign of Energy-related Products’ (MEErP) (Kemna, 2011), which consists of a techno-economic-environmental assessment structured on a series of tasks. This is the step where most of the technical challenges discussed in Section 2 arise and are subsequently addressed.

The next step of the Ecodesign process is the policy impact assessment (Step 2). Various policy options are analysed in this step against cost competitiveness, impact on small and medium enterprises, technological development and innovation, product functionality, and end-user affordability (European Commission, 2015c). The aim of the impact assessment of the potential policy is to provide an estimation of the potential benefits (in terms of resource savings, cost savings, industry competitiveness, etc.) of the improvement in the environmental performance of the product. As part of this assessment, a Consultation Forum meeting is convened in order to present proposals concerning the Ecodesign requirements to the stakeholders. Once the impact assessment has been successfully finalised, a draft of the potential Ecodesign regulation (step 3) is formally discussed in the EC (this process is known as ‘interservice consultation’). After that, notification of the draft Ecodesign regulation is sent to the World Trade Organisation (WTO). The draft Ecodesign regulation is then voted on by the EU member states in a Regulatory Committee (step 4). Following the three-month scrutiny period by the European Parliament and by the Council, Ecodesign regulations are finally published in the Official Journal of the European Union (Step 5).

The publication of an Ecodesign regulation for a certain product group may be followed by mandates to the European Standardisation Organizations (ESO) to develop standard methods for the assessment of the regulated parameters. It is worth noting that not all of the steps in the EU Ecodesign implementation process are open to stakeholders. Indeed, stakeholders typically provide inputs in the product’s preparatory study (step 1) and the policy impact assessment (step 2). Of the various stakeholders, it is important to distinguish the EU Member States Experts who are part of the regulatory committee and so have the final say on approval of the regulations (step 4).

Alt-text: Box 1

As discussed in Section 2, until now EU Ecodesign requirements have mainly targeted energy efficiency. CE strategies such as reparability, durability, upgradability, recyclability, or the identification of certain materials or substances will be systematically examined in the future (European Commission, 2015b). The development of material efficiency requirements for product policies should be prioritized in line with the more prominent role of CE in the EU policy agenda. The improvement of product design making the use of raw materials more efficient has been recognized as an EU policy interest in the past (European Commission, 2011). Since then, the Directorate General (DG) of the Joint Research Centre (JRC), the EC’s science and knowledge service, has been developing life-cycle environmental indicators related to material efficiency using a method called “Resource Efficiency Assessment of Products” (REAPro) (Ardente and Mathieux, 2012, 2014). This research has already inspired several revisions of the MEErP, which have been carried out by (Mudgal et al., 2013) with the introduction of CE aspect indicators into the MEErP, such as recyclability benefit rates, recycled content, and lifetime. However, this revision of the methodology has not been enough to push the development of more material efficiency requirements forward, mainly because of the challenges already discussed in Section 2 and more especially due to the timely involvement of relevant stakeholders.

In 2015, the DG JRC and the DG GROW^2^ started collaborating in research for the purpose of investigating further integration of material efficiency aspects into EU Ecodesign regulations. This collaboration developed by building on the respective knowledge bases and experiences of both organisations: the research on resource efficiency assessment carried out by DG JRC and the experience matured by DG GROW in implementing the Ecodesign Directive for several product groups. In particular, this collaborative research focused on the policy process to develop EU Ecodesign requirements for the ‘enterprise server’ product group.

This paper presents a critical analysis of this case-study experience (from 2015 to 2018) starting from the development of a more general concept of CE aspects and continuing up to the approval of concrete material efficiency policy measures. Challenges encountered during the various policy steps are analysed including solutions adopted and methods implemented. Lessons learnt from this case study are then used to suggest novel strategies to ‘operationalize’ CE principles into the Ecodesign product policy. With its concrete applicative nature from conceptualization up to the adoption in legislation, this research represents a ‘first-of-a-kind’ experience in the EU and worldwide that led to the identification of ambitious and measurable mandatory requirements now formulated in draft EU regulation.

## 4 Case study: the development of material efficiency requirements for enterprise servers

This section illustrates an analysis of CE aspects in a real-world Ecodesign implementation policy process: the development of the Ecodesign requirements for enterprise servers. Enterprise servers are computers used for business-to-business applications, e.g. typically in data centres or a company’s server room. These products were investigated in the context of the Ecodesign directive for reasons of their market penetration and their environmental impact. First, increasing market projections (related to trends in the Information and Communications Technology (ICT) sector such as the Internet of Things^3^, the Industry 4.0^4^, and Cloud Computing^5^) suggest that the environmental impact of these products will increase in the short to medium term. Secondly, as demonstrated in the supporting study (van Elburg et al., 2011) for the Ecodesign Working Plan 2012-14 (European Commission, 2012), these products could also potentially involve a significant environmental saving from the material efficiency point of view. Based on the above, the determinants of the environmental impact of enterprise servers, with specific regard to the CE aspects, have been analysed and discussed in previous papers (Talens Peiró and Ardente, 2015). Details about the material composition of enterprise servers are provided in the Supplementary Materials.

As an effect of their inclusion in the Ecodesign Working Plan 2012-14 (European Commission, 2012), enterprise servers were selected as a product to be subject to the Ecodesign implementation process. Material efficiency measures were addressed from the earliest stages of the preparatory study (step 1), which started in June 2013, in order to cope with the technical challenges to improve the presence of CE in product policies. Indeed, it was decided to pair the Ecodesign preparatory study^6^ (Berwald et al., 2015) with a specific material efficiency analysis conducted by the DG JRC (Talens Peiró and Ardente, 2015). The set of proposed CE requirements for enterprise servers is the main result of the technical analysis carried out by the authors of the latter study. Starting from an analysis of the environmental hotspots for the product group under analysis, the proposed requirements were identified as a response to these hotspots. Products compliant with these requirements will improve their material efficiency performances, overcoming the previously identified hotspots. After this first phase, in October 2015 a dedicated policy study, named as ‘impact assessment’, was started with the aim of in depth identification and development of the best regulatory solution for enterprise servers. Different policy options were investigated across different impact dimensions (economic, social, and environmental) (), and further data on the material efficiency aspects was gathered and analysed. The potential material efficiency requirements for CE were discussed with the stakeholders at the Consultation Forum meeting in the first quarter of 2017, and the feedback from this consultation helped to improve the formulation of the requirements, as discussed in the remainder of this section. All things considered, in the context of the initiative on the Ecodesign of servers, a wide range of consultations took place for the purpose of ensuring that the interests of all relevant sectors as well as citizens, non-governmental, and standardization organizations were duly taken into account. The feedback from stakeholders significantly contributed to the data gathering and analysis process.

In June 2018, the draft regulation of the Ecodesign of enterprise servers was made publicly available (European Commission, 2018b), allowing stakeholders a further month for specific and detailed comments on the draft legislative text. The Regulatory Committee vote in step 4 (see the Ecodesign implementation process of Fig. 2) took place in mid-September 2018 and, finally, the Ecodesign Regulation on enterprise servers was published in the Official Journal of the European Union in March 2019 (European Commission, 2019). The regulatory process has reached its full conclusion, which represents a great achievement especially considering the novel aspects involved in introducing the material efficiency requirements.

Of all of the possible aspects related to material efficiency, the discussions were narrowed to three main aspects: a) material composition of enterprise servers; b) recycling of servers at end of life; and c) the potential for reuse and remanufacture of servers. The discussion on these topics resulted in the proposal of four potential requirements concerning:

1) the design for disassembly of key-components (covering aspects b and c);
2) the declaration of content of CRMs^7^ (covering aspects a and b);
3) the provision of the latest available version of firmware (covering aspect c);
4) the availability of built-in functionality for secure data deletion (also covering aspect c).

A fifth additional requirement on the reusability of parts in servers initially proposed by Talens Peiró and Ardente (2015) was dropped^8^ during the steps of the process that followed.

Table 1 shows the formulations of these five material efficiency requirements for the CE of enterprise servers at steps 1, 2, and 3 of the Ecodesign implementation process.

**Table 1:** Comparison of the formulations of the material efficiency requirements for circular economy at steps 1, 2 and 3 of the Ecodesign implementation process.

	Formulation of material efficiency requirements at different steps of the Ecodesign policy process of enterprise servers		
	*In the JRC study (step 1)*	Discussed at the Consultation Forum (step 2)	In the draft Regulation (step 3)
***Requirement 1: Design for disassembly***	Manufacturers shall ensure that servers are designed so that external enclosures can be removed by hand or with commonly available tools. The following four types of components (when present) shall be identified, accessible, and removable by hand or with commonly available tools: • printed circuit board assemblies, including memory cards (larger than 10 cm^2^); • batteries; • hard disk drives; • the processor. The abovementioned components shall be: • accessible: this shall be ensured by documenting the sequence of disassembly operations needed to access the targeted components, including the following information for each operation: type of operation, type and number of fastening technique(s) to be unlocked, and tool(s) required; • extractable: servers shall be designed so that no fastening by welding or gluing is encountered for all of the disassembly operations leading to the extraction of the above-listed components (some exemptions should be foreseen for thermal paste and adhesives used to bind heat sinks to printed circuit boards).	From 1 January 2019, manufacturers shall ensure that welding or firm gluing is not used as joining or sealing technique for the following types of components, when present: (a) HDD and SSD (b) Memory (c) Processor (CPUs) (d) Motherboard (e) Chassis (f) Expansion cards/graphic cards (g) Power supply Accessing components shall be ensured by documenting the sequence of dismantling operations needed to access the target components, including the following information about each of these operations: type of operation, type and number of fastening technique(s) to be unlocked, and tool(s) required.	From 1 March 2020, manufacturers shall ensure that joining, fastening, or sealing techniques do not prevent the disassembly of the following components, when present: (a) data storage devices; (b) memory; (c) processor (CPU); (d) motherboard; (e) expansion card/graphic card; (f) power supply. Instructions on the disassembly operations referred to (…) of this Appendix, including the following information for each necessary operation and component: (a) the type of operation; (b) the type and number of fastening technique(s) to be unlocked; (c) the tool(s) required.
***Requirement 2: Statement of the content of CRM***	Information about the location and quantities of CRMs, especially rare earth elements contained in hard disks (HDDs), shall be provided by manufacturers for each product family architecture. This information shall be submitted to a centralised database organised by industry to consolidate the volume of CRMs in servers, which will provide reports or be accessible to recyclers or their representative organisations.	Total weight per product of the following critical raw materials if present, and indication of the components in which the following critical raw materials are present: (a) Cobalt, expressed in grams rounded to the nearest integer; (b) Neodymium, expressed in grams rounded to the nearest integer; (c) Palladium, expressed in grams to one decimal place	Weight range (less than 5 g, between 5 g and 25 g, above 25 g) of the following critical raw materials at component level, if present: (a) Cobalt in the batteries; (b) Neodymium in the HDDs;
***Requirement 3: Provision of the latest available Firmware***	The latest version of firmware to test the functionality and compatibility of different components in the server shall also be available	The latest version of firmware to upgrade and test the functionality and compatibility of the various components in the server shall be made available.	The latest available version of the firmware shall be made available for a minimum period of eight years after the placing on the market of the product, at a fair, transparent and non-discriminatory cost.
***Requirement 4: Availability of secure data deletion***	Data deletion of potentially reusable data storage equipment (i.e. hard disk drives, memory cards) shall be ensured by using the methods described in section 3.2.1.3 of this report.	Data deletion from potentially reusable data storage equipment (i.e. hard drives and solid state drives) shall be made possible by securing availability of built-in software based data deletion tool(s). Information on the data deletions tool(s) referred to in (…)	Built-in functionality for secure data deletion shall be made available for the deletion of data contained in all the data storage devices of the product. Information on the secure data deletion built-in functionality (…) including instructions on how to use the functionality, the techniques used, and the supported secure data deletion standard(s), if any;
***Requirement 5: Reused parts in enterprise servers***	*The annual total energy consumption of servers, reusing at least the HDD, memory card, CPU, and motherboard, shall not exceed the value* *“δj* ∙ E_TEC” (in kWh/year). *δj will have a value of between 7% and 20% depending on the number of components reused.*	*(discontinued)*	*(discontinued)*

Table 1 shows how well the changes in the formulations of the material efficiency requirements for enterprise servers in the Ecodesign implementation process, in particular in the first three steps, improved these requirements. It should be noted that steps 4 and 5 are the most ‘political’ ones where the amount of technical modifications to the legislative act is typically not significant. Indeed, the formulation at step 1, i.e., that resulting from the scientific work (Talens Peiró and Ardente, 2015), was correct from the technical point of view. However, some amelioration in terms of legal drafting was required as well as for the enforceability of the requirements, i.e., the verifiability of compliance by market surveillance authorities. The feedback from stakeholders, in particular industry, was also instrumental for the identification of less burdensome formulations of the requirements. Finally, the policy analysis carried out under the ‘impact assessment’ confirmed that the proposed material efficiency requirements were justified in environmental-economic terms. Indeed, the CE principles could be “operationalized” in terms of Ecodesign requirements for enterprise servers thanks to both:

- the robust technical analysis supporting the policy (mainly in step 1, but also continuously throughout the whole process), in which the CE aspects were systematically identified, analysed, and discussed with solid methodological grounds;
- the timely and continuous involvement of relevant stakeholders, together with policy makers and material efficiency experts.

More in detail, the evolution of the formulation of the requirement on design for disassembly (from step 1–2) resulted in the proposal of a clear and enforceable requirement. Indeed, the requirement as drafted at the Consultation Forum stage (i.e. step 2 in Table 1) would have banned welding or firm gluing as joining techniques for certain components such as batteries, HDDs, or processors. Although these techniques were recognized as critical in fostering the repair of enterprise servers as well as being effective in improving recycling, OEM in particular (Digital Europe, 2017) claimed that the ban on these joining techniques could hamper future innovation and competitiveness in the IT industry. Following discussions at the stakeholder consultation forum, the requirement was reverted into (from step 2 to step 3) a more technologically neutral formulation.

The changes in the material efficiency requirement 2 on the presence of CRM show how the requirement was streamlined on the one hand for the purpose of providing useful information to recyclers and on the other hand not overburdening OEM. So requirement 2 was defined using the feedback from OEM, recyclers, and market surveillance authorities. The final version of requirement 2 considers a limited number of materials (i.e. Neodymium and Cobalt) and components (batteries and HDDs), also prescribing a weight range (i.e. less than 5 g, between 5 g and 25 g, above 25 g) instead of the specific weight which was judged by industry to be very burdensome, and difficult for the market surveillance authorities to enforce.

The main change in the formulation of requirement 4 on secure data deletion of the information contained on a HDD is the opportunity for manufacturers not only to provide data deletion functionality via pre-installed software on the machine but also using other technical solutions such as pre-installation in the firmware (typically in the Basic Input/Output System) or also in software included in a self-contained bootable environment (such as an USB drive). The final version of this requirement was developed in response to the comments of the industrial stakeholders (Digital Europe, 2017). While keeping the rationale and the aims of this specific requirement unchanged, the resulting requirement ensures manufacturers can take a more flexible approach.

Repairers vocally supported requirement 4 on the availability of the latest firmware while OEM (Digital Europe, 2017) claimed that financial compensation is necessary. Finally, consensus was built around the compromise that OEMs have to provide the latest available version of the firmware at a ‘fair, transparent, and non-discriminatory cost’. While the expression ‘fair, transparent, and non-discriminatory’ cost is already present in EU law (European Commission, 2016b), it is possible that some market surveillance authorities will judge this formulation to be difficult to verify and enforce. If needed, guidance documentation could be produced in order to help with this specific aspect. The requirement on firmware availability is expected to be the most effective in fostering the repair of enterprise servers (Polverini et al., 2018). Overall, researchers and policy makers have made a constant effort throughout the whole process to ensure actual enforceability of the proposed requirements on material efficiency. The situation referred to in the case of the requirement on 'fair, transparent, and non-discriminatory cost', i.e., the potential need for guidance documentation, is a rather peculiar case. In the context of this ‘first-of-a-kind’ experience on mandatory CE requirements, the proposed formulation of the requirement on firmware availability was judged to be the best trade-off between, on the one hand, the expected effectiveness of this requirement and, on the other hand, an obligation on manufacturers that is not excessively burdensome.

In conclusion, potential material efficiency requirements for CE were described thanks to the close and continual interaction with the stakeholders, and to the collaborative work in parallel, the Ecodesign implementation process for the enterprise server product group. This experience was the first of its kind to include scientific and technical data input and a quantitative assessment of material efficiency aspects in the early steps. Indeed, starting the discussion at the beginning of the Ecodesign implementation process allowed^9^ the development of the four original material efficiency requirements which are part of the final version of the regulation voted by the EU Member states. A summary of the of the main steps for the implementation of CE strategies in the development of Ecodesign requirements for enterprise servers is presented in the Supplementary Materials (Table S2).

## 5 Discussion: a novel policy approach to progressing towards a circular economy in EU product policy

The steps in the EU Ecodesign implementation process (see Fig. 2 and Table S2 in The Supplementary Materials) with the greatest technical and practical challenges were identified. Most of the technical challenges arise in the preparatory study (step 1) and the policy impact assessment (step 2). Indeed, Fig. 2 is completed with a series of icons that represent the type of inputs proposed that would partially help to overcome the technical challenges discussed in Section 2. Each icon represents a type of information provided during the policy implementation process. A ‘people icon with M’ represents the stakeholder input in the field of material efficiency and CE. A ‘database icon’ illustrates the scientific and technical data input. A ‘histogram icon’ represents quantitative assessments that allow metrics to support MEErP on material efficiency aspects. Material efficiency indicators from JRC could also be used to support scientific and technical data as well as provide a quantitative assessment and support MEErP. For instance, Table S1 in the Supplementary Materials includes an example of the recyclability benefit indicator, which expresses the potential environmental savings that can be achieved from recycling the product over the environmental burdens of virgin production followed by disposal. To support a more comprehensive and scientifically based debate about material efficiency measures for the EU CE, the authors suggest that detailed technical evidence is collected during the product preparatory study (step 1) and the policy impact assessment (step 2).

Following a bottom-up approach, five novelties have been identified to address the challenges identified in Section 2.

• ***Novelty 1: to ensure early (preferably during the product preparatory study, step 1) interaction between policymakers, material efficiency experts, and other stakeholders specialised in the product group under analysis.***
• ***Novelty 2: to have a policy process fully equipped with relevant material efficiency data inputs and indicators.***

The additional information on material efficiency aspects gathered thanks to stakeholder meetings and consultations is extremely valuable to the scope of the analysis. Inputs collected from manufacturers, independent IT service providers, end-of-life operators, and ESOs contribute to both the data gathering process and to increasing the robustness, effectiveness, and acceptability of requirements for the approval of the draft policy proposal. The approach proposed suggests that the intensity of quantitative assessments should be gradually increased throughout the process. The more available data on the product group and on the potential end-of-life scenarios is, the more consensus on the relevance of a given material efficiency requirement, and the greater the chances of material efficiency requirements being included in newer EU Ecodesign product policies. Including information proposed in this new approach is in line with the conclusions from Bundgaard et al. (2017). The consultation forum included in the policy impact assessment (step 2) represents a good step to fine-tune material efficiency requirements, and the related methods to verify the proposed requirement. This leads to another novelty of the proposed approach:

• ***Novelty 3: to maintain a continuous and iterative process based on multiple stakeholder consultations.***

The detailed analysis of material efficiency aspects of enterprise servers represents a sound evidence base, which will be of fundamental help in the legislative process. In terms of collecting the inputs from stakeholders, the industrial ones (i.e. the manufacturers) certainly had the most important role. On one hand, having required their input on multiple occasions, and on the other hand, shared the data and analyses in the stakeholder meetings for the preparatory study as soon as they became available, was certainly helpful. It was also crucial to involve new types of stakeholders (e.g. recyclers, reuse and refurbishing companies) during the course of the analysis whose participation has been generally lacking in other Ecodesign processes. This is in line with the findings of other authors about the relevance of identifying stakeholders related to the whole supply chain of goods and services investigated (Fritz et al., 2018). Furthermore, thanks to the involvement of material efficiency experts, it was possible to identify and tackle the “lack of knowledge” encountered during the policy process in a timely manner, for example, concerning the collection of technical evidence about the recycling and reuse of servers. Although the importance for involving recyclers and other material efficiency experts in Ecodesign policy process had already been highlighted by some authors, e.g. (Ardente et al., 2015), the server product group was the first policy process in which these kinds of stakeholder were involved from the very early stages. This leads on to the next novelty of the proposed approach:

• ***Novelty 4: to involve material efficiency experts (including recyclers, re-use operators, etc.) during the whole process, including early stages.***

The Ecodesign regulation on enterprise servers was finalized in March 2019 (European Commission, 2019). However, there have already been clear reactions from diverse stakeholders. Some reuse operators are in favour of the concrete CE policy requirements but OEMs are not fully supportive of potential material efficiency policy measures (Digital Europe, 2017). Such scepticism is also in line with previous studies (Dalhammar et al., 2014; Bundgaard et al., 2017). The main criticism from OEMs is that enterprise servers are business-to-business products, and, given the significant investments required when purchasing these products, most OEMs have already well developed take-back and asset recovery schemes, e.g., within service provision contracts. Some examples of these take-back schemes are the ‘Global Asset Recovery Services’ (IBM, 2019), the ‘IT Asset Lifecycle Solutions’ (HP, 2019), and the ‘Dell Asset Recovery Services’ (Dell, 2019). The lack of common definitions and technical descriptions of these existing server recovery services hindered comparison of the systems implemented by many OEMs. According to OEM associations, this proves that it is in their own interest that the enterprise servers are designed for efficient maintenance, allowing the products to be repaired, updated, and reconfigured. However, not all OEMs have implemented such virtuous practices.

The present research confirmed that servers have high reuse and recycling rates. However, in contrast to the arguments brought by OEMs, the analysis revealed that a significant share of servers (around 15%–20%) do not actually return to OEMs, being collected and treated by other stakeholders (such as refurbishing companies, spare parts providers, and WEEE recycling plants). Moreover, the analysis carried out with some end-of-life operators proved that not all the servers are optimally designed for repair and re-use by third parties. Relevant information for recycling is not systematically provided by all of the OEMs for all product models. While reuse companies pointed out the unavailability of firmware as the major obstacle to reusing servers, OEMs were concerned about the provision of firmware as it is generally strictly proprietary.

• ***Novelty 5: to develop material efficiency requirement based on available (or under development) standardised methods.***

Another barrier encountered during the process was the lack of standardised methods for the assessment of the material efficiency requirements. Requirements need to be measurable (ideally with standards) in order to be enforceable by market surveillance authorities. The lack of standards is an area of “lack of knowledge” according to the representation in Fig. 1. For example, to date there is no standardised method to describe the sequence of dismantling operations of electr(on)ic equipment nor assessing their ease of disassembly. Research work on this topic is currently on-going (Vanegas et al., 2018) and could be fed into EU horizontal standards on material efficiency aspects currently under development (European Commission, 2015b) or, if more granularity of the analysis were available, under a dedicated standardisation mandate on the Ecodesign of enterprise servers. Standards developed under this latter mandate would be built on the basis of the general methods laid down in the horizontal standards on material efficiency aspects. This need for standardisation is also in line with outcomes of other studies in the literature (Dalhammar et al., 2014; Bundgaard et al., 2017; Tecchio et al., 2017). In the case of enterprise servers, requirement 1 on design for disassembly was formulated to be verifiable despite the current absence of standards on the assessment of ease to disassemble. On the other hand, the presence of ‘first-of-a-kind’ material efficiency requirements for CE could stimulate the standardisation process. For example, the requirement on the content of certain CRMs in the servers is aimed at improving knowledge of product composition and its trends over the time, and consequently to encouraging more efficient recycling in the future. Following the example of this requirement, the recent standard EN 45558 has further defined and detailed how information on CRMs in the products could be practically collected and communicated by manufacturers (including information from the supply chain). On the other hands, initial drafts of the standard EN 45558 were used by policy makers to refine the formulation of the requirement on CRMs.

The comments discussed so far clearly show the essence of the consultation process: stakeholders engage in the discussion putting forward their viewpoint on technical and policy aspects, trying to influence the decision towards more favourable conditions for them. Considering the schematization of the policy process in Fig. 1, this case study was indeed characterized by the participation of heterogeneous stakeholders with very different objectives, and by the continuous “pull” by the policymakers towards, among other things, the “circular economy” target (not forgetting more “traditional” targets such as energy efficiency).

The novel elements presented in this paper will have to undergo further testing in the context of Ecodesign by applying them in other product groups in order to refine and streamline them. Moreover, although the proposed approach has been formulated in the context of the Ecodesign Directive, it could also be valuable for other product policy instruments such as the EU Ecolabel Regulation (European Parliament and the Council of the European Union, 2010) and Green Public Procurement (GPP) (European Commission, 2008) to foster the CE product performances. Material efficiency requirements in those cases are likely to be more restrictive as the objective is shifted towards increased environmental sustainability. In the EU Ecolabel and GPP, the goal is to identify environmental excellence among the existing products while Ecodesign aims to restrict the access of the worst performing products to the EU market.

## 6 Conclusions

This article discusses a case study that demonstrates how CE strategies are enabled in European product policy. The lessons learnt during this case study help formulate a novel approach to “operationalise” CE principles into product policy. The results of this analysis are of general interest whenever an effective implementation of CE into product policy is needed, and applying the approach to a case study proved to be effective.

The main novel features of the proposed approach were: 1) consideration of CE aspects and objectives in the early stages of the policy process; 2) use of dedicated (and innovative) metrics to measure material efficiency along the process; 3) continual collection of detailed technical evidence to support material efficiency assessment and iterative discussions throughout the process; 4) the enlarged involvement of relevant stakeholders generally not present during the Ecodesign stakeholder meetings (such as reuse and repair centres, and recyclers); 5) the push towards the development of relevant standards. The successful application of the approach was facilitated by close collaboration between EU policy officers and material efficiency experts with experience in policy formulation. In particular, to the authors' knowledge, the case study experience of enterprise servers represented the first example in Europe and one of the first examples in the world in which the reusability of the products and the composition of the product in terms of CRMs have been assessed in detail and successfully implemented in mandatory policy measures. The proposed material efficiency measures for enterprise servers are also original as for the first time they combine the minimum requirements for the hardware and physical aspects (e.g. requirement 1 on design for disassembly), the documentation requirements (e.g. requirement 2 on presence of CRMs), and the minimum requirements for software aspects (e.g. requirement 3 and 4 on firmware and secure data deletion).

On the other hand, the main limitations of the approach were:

- conflicting interests and objectives of stakeholders, which can potentially hinder the general consensus;
- legal problems applying to specific issues (e.g. liability for the distribution of certain information; proprietary knowledge);
- a lack of a detailed picture of all the flows of waste servers within all the EU member states;
- a lack of specific figures on the size of investments for recyclers;
- the full verifiability of certain requirements (for example, due to the lack of standards to describe the sequence of dismantling operations).

Further tailored policy innovations (e.g. additional metrics to assess circularity strategies, such as remanufacturing (Ardente et al., 2018); standardised metrics to measure performances of products) and experience with several product groups will probably be necessary to overcome these limitations.

In conclusion, albeit with some limitations, this experience and the novel approach proposed is very capable of enhancing the implementation of CE strategies in a product policy instrument like the Ecodesign Directive. The legislative work on enterprise servers was finalized in March 2019 (European Commission, 2019) once again confirming the robustness of the technical analysis behind the policy. The experience on the enterprise servers product group triggered the general interest of many actors in the material efficiency requirements for CE and their related impacts and will also contribute to a more systematic emphasis on CE aspects in future product requirements under the Ecodesign Directive.

## Disclaimer

The views expressed in the article are personal and do not necessarily reflect an official position of the European Commission. Neither European Union institutions and bodies nor any person acting on their behalf may be held responsible for the use that may be made of the information contained therein.

## Footnotes

1 Here the term “policymaker” refers to people responsible for formulating policies (whatever the regulated field).

2 Directorate General for Internal Market, Industry, Entrepreneurship, and Small and Medium Enterprises.

3 The Internet of Things (IoT) represents the next step towards the digitalisation of our society and economy, where objects and people are interconnected through communication networks and report about their status and/or the surrounding environment.

4 The current trend of automation and data exchange in manufacturing technologies.

5 The practice of using a network of remote servers/storage devices hosted on the Internet (rather than a local server or a personal computer) to store, manage, and process data.

6 The preparatory study also tackled the assessment of data storage devices but these were not discussed in this paper.

8 This requirement was rejected because while conceptually and methodologically correct, it would not have been practically enforceable for the two main reasons. First, at the time when the technical analysis was carried out by Talens Peiró and Ardente (2015), no method for the evaluation of the total energy consumption (TEC) of a server was available. Moreover, the correct enforcing of requirements on reused components would involve the need for traceable and reliable information, e.g., at the level of the bill of material for the product and/or in the supply chain in order to identify and trace the reused components. This kind of information is still not available for enterprise servers marketed in the EU (as well as for most other products).

9 The preparatory study also formulated several potential measures concerning energy efficiency such as the efficiency requirements for the internal power supply unit; requirements for product performance (both in idle and active state); and requirements on product operating conditions (mainly: regarding operation at higher temperatures).

## Footnotes

7 Securing reliable and unhindered access to certain raw materials is a growing concern within the EU and across the globe. To address this challenge, the European Commission has created and continuously updated a list of materials that are recognized as ‘Critical Raw Materials (CRMs)’ for the EU, i.e., raw materials that are of great importance to the EU economy and have a high risk associated with their supply (European Commission, 2017).
