# Supplementary material for: Advances towards circular economy policies in the EU: The new Ecodesign regulation of enterprise servers
Source: Resour Conserv Recycl. 2020 Mar;154:104426. doi: 10.1016/j.resconrec.2019.104426 (PMC7015273; doi:10.1016/j.resconrec.2019.104426)
Supplement: Supplementary file 1 [file mmc1.pdf]

Supplementary Materials:

Advances towards circular economy policies in the EU: the new Ecodesign regulation of enterprise servers

Laura Talens Peiró[1], Davide Polverini[2], Fulvio Ardente[3], Fabrice Mathieux[3]

[1] Beatriu de Pinós at Sostenipra, Institut de Ciencia i Tecnologia Ambientals (ICTA-UAB), Universitat Autònoma de Barcelona, 08193 Cerdanyola del Vallès, Barcelona, Spain. (Cerdanyola del Vallès), Barcelona, Catalonia, Spain
[2] European Commission, DG Internal Market, Industry, Entrepreneurship and SMEs, Brussels (Belgium)
[3] European Commission, Joint Research Centre (JRC), Ispra (Italy)

The material composition of enterprise servers, also known as the bill of materials (BOM), is crucial in estimating the content of materials in servers, performing a life cycle assessment (LCA), and calculating material efficiency indicators. Data for the BOM was partially taken from data of the Ecodesign preparatory study (Berwald et al., 2014), and from two exemplary servers dismantled by the JRC during the study (Talens Peiró and Ardente, 2015). Among the parts contained in the servers, printed circuit boards (PCBs) and hard disk drives (HDDs) were especially targeted as they contained several materials included on the EU list of raw materials (EC, 2017).

The JRC's focus for the research was on reuse and recycling and on the design of the servers. An important finding was that some components (i.e. hard disk drives, memory cards, the central processing unit, and the motherboard) could be easily extracted and reused for new and remanufactured servers. Two material efficiency indicators were used to estimate the potential environmental benefits of reuse and recycling: the reusability benefit rate and the recyclability benefit rate (Ardente and Mathieux, 2014b). It was concluded that servers reusing certain parts are generally environmentally preferable compared to servers with only new components. For example, reusing certain components (e.g. hard disk drives and memory cards) is environmentally beneficial even if the remanufactured server consumes up to 7% more energy than a newly manufactured server (Ardente et al., 2018). This value rises to 20% when the number of reused components increases (including also CPUs and motherboard) (Ardente et al., 2018).

Addressing material efficiency requires appropriate metrics. The method developed by JRC was used to provide numerical results for two recycling scenarios: the combination of manual and automatic recycling treatments, and recycling using unsorted shredding. Although the differences of the results for both scenarios are small in terms of overall recycled mass (62% and 60% respectively), there are still significant differences for some minor and critical metals included on the printed circuit board (PCBs). For instance, the recycling amount of gold, palladium, and silver falls from 98-99% to 11-26% when unsorted shredding occurs.

Table S1. Recyclability benefit rate for enterprise servers for the combination of manual and automatic recycling treatment.

Impact category	Indicator	Unit	Recyclability Benefit Rate
Ecotoxicity for aquatic freshwater	USEtox (recommended)	[CTUe]	67%
Freshwater eutrophication	EUTREND model, ReCiPe	[kg P eq]	65%
Human toxicity cancer effects	USEtox (recommended)	[CTUh]	15%
Human toxicity non-cancer effects	USEtox (recommended)	[CTUh]	14%
Climate change	IPCC global warming, excl. biogenic carbon	[kg CO_2-Eq.]	1%
Climate change	IPCC global warming, incl. biogenic carbon	[kg CO_2-Eq.]	1%
Marine eutrophication	EUTREND model, ReCiPe	[kg N-Eq.]	3%
Ozone depletion	WMO model ReCiPe	[kg CFC-11 Eq.]	37%
Particulate matter/Respiratory inorganics	RiskPoll	[kg PM2,5-Eq.]	8%
Photochemical ozone formation	LOTOS-EUROS model, ReCiPe	[kg NMVOC]	5%
Terrestrial eutrophication, accumulated exceedance		[Mole of N Eq.]	4%
Total freshwater consumption, including rainwater	Swiss Ecoscarcity	[UBP]	1%
Abiotic Depletion (ADP elements)	CML2001 - Apr. 2013	[kg Sb-Eq.]	63%
Abiotic Depletion (ADP fossil)	CML2001 - Apr. 2013	[MJ]	1%
Acidification Potential (AP)	CML2001 - Apr. 2013	[kg SO_2-Eq.]	10%
Primary energy demand	ren. and non ren. resources (gross cal. value)	[MJ]	1%

Table S1 shows the recyclability benefit rate for enterprise servers for the combination of manual and automatic recycling treatments compared to unsorted recycling. This indicator estimated the environmental benefits (from a life cycle point of view) that can be achieved from the recycling of a product at the end of life, calculated on the basis of the environmental impact assessment categories used in the Life Cycle Assessment methodology (Ardente and Mathieux, 2012). The environmental benefits of the combined recycling scenario are very high (over 60%) for impact categories such as the ecotoxicity of aquatic freshwater, freshwater eutrophication, and abiotic depletion of elements. The potential savings of the combined recycling scenario in terms of human toxicity, acidification, and particulate matter are respectively about 15%, 10%, and 8% of the life cycle impacts. However, the implementation of the combined recycling scenario (and of related benefits) requires several preconditions to be met. Firstly, waste should be collected carefully and delivered to the plant, avoiding damage that might hamper dismantling. Secondly, recycling operators should be trained in the architecture of the server, the location of key components, and their dismantling. Moreover, the recycling plant should be equipped with different lines for the treatment of the various dismantled

components (e.g. cables, large plastic parts, electronics), whereas mechanical treatments should be applied to components that can be efficiently separated after shredding (e.g. metal parts) or complex components that cannot be easily dismantled manually (e.g. complex plastic parts).

A synoptic table of the development process of Ecodesign requirements for enterprise servers is presented in Table S2, the steps being numbered in the same way as in Figure 2 of the paper. Table S2 provides a chronological overview of the most significant activities from the material efficiency point of view, starting with the inclusion of this product group in the Ecodesign Working Plan 2012-2014 (EC, 2012), continuing with the preparatory study, and moving on to September 2018, with the meeting for the approval of the draft Ecodesign regulation by the Regulatory Committee (step 4). The conclusion of the process is shown in the next line, with the publication of the Ecodesign Regulation on enterprise servers on the Official Journal of the European Union. The fourth column of Table S2 especially focusses on how material efficiency has been tackled in this real policy process. Relevant horizontal initiatives in the field of circular economy and policy are also listed, such as the publication of the Circular Economy Action Plan (EC, 2015a). The synoptic table also gives an estimate of the timing of the next steps (such as the adoption of the Ecodesign Regulation on enterprise servers by the EC). Table S2 also references the final steps, which could presumably take place toward the end of the process (e.g. the potential launch of standardisation activities).

Table S2. Summary of the most significant steps for the implementation of circular economy strategies in development of Ecodesign requirements for enterprise servers

No	Step	Date	'Vertically'[1] relevant material efficiency aspects	'Horizontally'[2] relevant material efficiency aspects
0	Ecodesign Working Plan 2012-2014	Dec. 2012		It mentions that "increased attention will be paid to the identification of Ecodesign requirements on material efficiency in forthcoming preparatory product studies and reviews, when these aspects are found to be significant"
1	Start of the "Ecodesign preparatory study on enterprise servers and data equipment"	June 2013	The preparatory study aims to assess the feasibility of (among other things) a potential Ecodesign Regulation to mitigate the environmental impact of enterprise servers and data equipment, typically by means of energy efficiency or circular economy requirements.	
1	First consultation questionnaire	Oct. 2013	Input: Contribution of industrial stakeholders on (among others) the current practices on reuse, repair and recycling of the product group in the EU	
1	First draft report (Tasks 1 to 4)	Dec. 2013	- Discussion of product lifetime<br>- Detailed analysis of end-of-life behaviour including reuse and repair	
1	Revision of the MEErP methodology	Dec. 2013		The December 2013 version of the MEErP addresses some material efficiency aspects (including recycling benefits, durability and recycled content). The revised MEErP was used for the first time for the study of enterprise servers.
1	1st Stakeholder meeting	Dec. 2013	- Adoption of revised MEErP methodology (2013) for the preparatory study<br>-Preliminary discussion of repair/refurbishment practices and their impact on product lifetime estimation	
1	Input from JRC about material efficiency aspects on servers	Feb. 2014	Material efficiency experts from the JRC join the technical discussion, based on preliminary assessments of material efficiency criteria (dismantling, recyclability, durability, etc.) on enterprise servers	
1	Second draft report (Task 1 to 5 of MEErP)	May 2014	- Detailed bill of materials (BOM) of servers, including critical raw materials content<br>- Detail of impacts of printed circuit boards to be used as input for the assessment<br>- Calculation of recyclability benefit rate	
1	2nd Stakeholder meeting	Oct. 2014	Policy makers and material efficiency experts highlight the importance of obtaining feedback from stakeholders on critical raw materials and recyclability issues	
1	Third draft report (Tasks 1 to 7)	Mar. 2015	Benefits of reuse stated based on JRC study results	
1	3rd Stakeholder meeting	Apr. 2015	Discussion on the potential of the proposed requirements for design of reuse and recycling	

[1] Aspects relevant to the specific product group "enterprise servers".
[2] Aspects relevant to all product groups.

Table S2. Summary of the most significant steps for the implementation of circular economy strategies in development of Ecodesign requirements for enterprise servers

No	Step	Date	'Vertically'[1] relevant material efficiency aspects	'Horizontally'[2] relevant material efficiency aspects
1	Final Preparatory study report	Sept. 2015	Potential Ecodesign requirements, based on JRC study, drafted including requirements on:<br>- Dismantling, reuse and recycling<br>- Critical raw material content<br>- Technical documentation on material efficiency aspects	
1	Circular Economy Action Plan	Dec. 2015		The EC launches its action plan to foster the transition from the traditional linear economic model based on a 'take-make-consume-throw away' pattern to more of a circular economy based on reuse, repair, and recycling. Ecodesign is identified as one of the legislative tools to deliver on these aspects. A mandate to develop standards for material efficiency aspects of products was also issued to ESOs (EC, 2015b).
2	Start of Impact Assessment	Oct. 2015	Impacts (economic impacts for businesses and resource savings) of circular economy requirements are assessed in detail	
2	Draft Ecodesign requirements at the Consultation Forum	Feb. 2017	Circular economy requirements were proposed: for disassemblability; for data deletion to facilitate reuse/repair; for information concerning critical raw materials content; on firmware availability	
3	Interservice consultation	April 2018	Proposal of circular economy requirements	
4	Regulatory Committee vote by the EU member states	Sept. 2018	Proposal of circular economy requirements	
5	Publication of the Ecodesign Regulation on enterprise servers	March 2019	Publication of circular economy requirements[3]	
	Possible standardisation mandate for enterprise servers	2nd quarter 2019*	A product specific standardisation mandate concerning material efficiency aspects could be launched in the second quarter of 2019. It would benefit from the horizontal standardisation mandate on material efficiency aspects (EC, 2015b).	
* estimated dates				

[3]See https://eur-lex.europa.eu/legal-content/EN/TXT/?qid=1553903370788&uri=CELEX:32019R0424

Table S3. BOM of an example rack server (Talens Peiró and Ardente, 2015).

Component	Material	Mass (g)	Component	Material	Mass (g)
**Chassis**	Steel	12 265	**Motherboard**	Controller Board	1 667
	Plastics (ABS)	348			
	Plastics (PC)	282	**2 PSUs**	*See table S4**	3 426
	Aluminium	249	**Expansion card/ other**	PCB	349
	Copper	179	**Cables**	Brass	7
	PCB	131		Copper	81
**4 Fans**	*See table S4**	946		Zinc	96
**4 HDDs**	*See table S4**	1 748		Plastics (HDPE)	104
**ODD**	Low alloyed steel	115		Plastics (PVC)	145
	Copper	7		PUR	2
	Aluminium	1		Synthetic rubber	35
	Plastics (HDPE)	28	**2 CPUs**	*	54
	Plastics (ABS)	12	**Heat Pipes for CPUs**	Low alloyed steel	140
	Plastics (PC)	7		Copper	442
	PCB	19	**Memory**	PCB	135
			**Packaging**	Cardboard	3 629
**Batteries**	CR2032 (button)*	1.6		Plastics (HDPE & other)	78
	Lithium ion (prismatic)*	43		Plastics (GPPS/ Styrofoam)	1 026
**TOTAL: 27,799 g**					

* Primary data from the disassembly of servers by the JRC. ABS: acrylonitrile-butadiene-styrene; PC: polycarbonate; HDPE: High density polyethylene; PUR: Polyurethane; PVC: polyvinyl chloride.

Table S4. Primary data of typical fans, HDDs, and PSUs obtained from JRC analyses (Talens Peiró and Ardente, 2015).

Component	Materials / Components	Mass (%)	Component	Materials / Components	Mass (%)
Fan	Steel	40.8	PSU	Steel	39.3
	Copper	8.2		Aluminium	6.6
	Iron based	5.8		Fan[1]	4.5
	Plastic (PBT-GF30)	21.8		Plastic (EVA)	2.2
	Plastic (PCABSFR40)	2.2		Plastic (PCFR40)	1.5
	Plastic (undefined)	21.1		PCB	45
				Cables	0.9

Component	Materials / Components	Mass (%)	Materials / Components	Mass (%)
HDD	Aluminium	45.0	Magnet	3.9
	Steel	31.3	PCB	3.9
	Ferrous based	8.7	Plastic (PCABS)	3.9
	Copper	0.4	Plastic (PCGF)	3.0

[1] The material composition is the same as that already included in this table under 'Fan'. EVA: Ethylene-vinyl acetate; PBT: polybutyl terephthalate; PCABS: polycarbonate acrylonitrile; PCABSFR40: polycarbonate acrylonitrile containing flame retardant; PCFR40: polycarbonate containing flame retardant.

Table S5. Estimated amount of critical raw materials in enterprise servers (Talens Peiró and Ardente, 2015).

Material	Symbol	Amount per server (mg)
Antimony	Sb	4,436
Beryllium	Be	35
Cobalt	Co	9,266
Magnesium	Mg	4
Palladium	Pd	397
Silicon [a]	Si	11,227
Rare earth elements (REEs)		
Dysprosium	Dy	3,604
Neodymium	Nd	14,629
Praseodymium	Pr	3,604
Terbium	Tb	748

[a] Silicon in servers is contained in different grades: electronic grade (9N) in the die of packages, and in stainless steel alloys

REFERENCES

Ardente, F. and F. Mathieux (2012). Integration of resource efficiency and waste management criteria in European product policies – Second phase Report n° 3 - Refined methods and Guidance documents for the calculation of indices concerning Reusability / Recyclability / Recoverability, Recycled content, Use of Priority Resources, Use of Hazardous substances, Durability (final), Joint Research Centre (JRC) Technical Report (available at: https://publications.europa.eu/s/jtlR accessed November 2018).

Ardente, F. and F. Mathieux (2014b). "Identification and assessment of product's measures to improve resource efficiency: the case-study of an Energy Using Product." J. Clean. Prod. 83: 126-141.

Ardente, F., Talens Peiró, L., MAthieux, F., Polverini, D., 2018. Accounting for the environmental benefits of remanufactured products: Method and application. J Clean Prod, 198, 1545-1558. https://doi.org/10.1016/j.jclepro.2018.07.012

Berwald, A., T. Faninger, et al. (2015). Ecodesign Preparatory Study on Enterprise Servers and Data Equipment. Luxembourg.

European Commission (2012). Commission staff working document: Establishment of the Working Plan 2012-2014 under the Ecodesign Directive, SWD(2012) 434 final.

European Commission (2015a). Communication from the commission to the European parliament, the council, the European economic and social committee and the committee of the regions 'Closing the loop - An EU action plan for the Circular Economy'. COM(2015) 614/2: 1-21.

European Commission (2015b). Commission implementing decision of 17.12.2015 on a standardisation request to the European standardisation organisations as regards ecodesign requirements on material efficiency aspects for energy-related products in support of the implementation of Directive 2009/125/EC of the European Parliament and of the Council. C(2015) 9096 final: 1-13.

European Commission (EC), (2017). Communication from the Commission to the European Parliament, the Council, the European economic and Social Committee and the Committee of the Regions on the 2017 list of Critical Raw Materials for the EU, COM/2017/0490 final.

Talens Peiró, L., Ardente, F., (2015). Environmental Footprint and Material Efficiency Support for product policy - Analysis of material efficiency requirements of enterprise servers. Publications Office of the European Union. doi: 10.2788/409022
